# Field-Portable Microplastic Sensing in Aqueous Environments: A Perspective on Emerging Techniques

**DOI:** 10.3390/s21103532

**Published:** 2021-05-19

**Authors:** Morgan G. Blevins, Harry L. Allen, Beckett C. Colson, Anna-Marie Cook, Alexandra Z. Greenbaum, Sheila S. Hemami, Joseph Hollmann, Ernest Kim, Ava A. LaRocca, Kenneth A. Markoski, Peter Miraglia, Vienna L. Mott, William M. Robberson, Jose A. Santos, Melissa M. Sprachman, Patricia Swierk, Steven Tate, Mark F. Witinski, Louis B. Kratchman, Anna P. M. Michel

**Affiliations:** 1 MIT-WHOI Joint Program in Oceanography/Applied Ocean Science & Engineering, Cambridge and Woods Hole, MA 02543, USA; mblevins@mit.edu (M.G.B.); bcolson@whoi.edu (B.C.C.); 2 Department of Applied Ocean Physics and Engineering, Woods Hole Oceanographic Institution, Woods Hole, MA 02543, USA; 3 Department of Aeronautics and Astronautics, Massachusetts Institute of Technology, Cambridge, MA 02139, USA; 4 The Charles Stark Draper Laboratory Inc., Cambridge, MA 02139, USA; azgreenbaum@draper.com (A.Z.G.); jhollmann@draper.com (J.H.); ekim@draper.com (E.K.); alarocca@draper.com (A.A.L.); kmarkoski@draper.com (K.A.M.); pmiraglia@draper.com (P.M.); jsantos@draper.com (J.A.S.); msprachman@draper.com (M.M.S.); pswierk@draper.com (P.S.); state@draper.com (S.T.); mwitinski@draper.com (M.F.W.); 5 Emergency Response Office, Superfund Division, U.S. EPA Region 9, San Francisco, CA 94105, USA; Allen.HarryL@epa.gov; 6 Department of Mechanical Engineering, Massachusetts Institute of Technology, Cambridge, MA 02139, USA; 7 Kamilo, Inc., Former U.S. EPA Region 9, San Francisco, CA 94108, USA; amc2037@gmail.com; 8 Department of Electrical and Computer Engineering, Northeastern University, Boston, MA 02115, USA; sheila.hemami@gmail.com; 9 Draper, Bioengineering Division, Cambridge, MA 02139, USA; vmott515@gmail.com; 10 Ocean P3 Systems, Former U.S. EPA Region 9, San Francisco, CA 94108, USA; bill@oceanp3systems.com

**Keywords:** microplastics, plastic pollution, sensors, analytical chemistry, environment, water, ocean, marine pollution, polymers, freshwater, aqueous solutions

## Abstract

Microplastics (MPs) have been found in aqueous environments ranging from rural ponds and lakes to the deep ocean. Despite the ubiquity of MPs, our ability to characterize MPs in the environment is limited by the lack of technologies for rapidly and accurately identifying and quantifying MPs. Although standards exist for MP sample collection and preparation, methods of MP analysis vary considerably and produce data with a broad range of data content and quality. The need for extensive analysis-specific sample preparation in current technology approaches has hindered the emergence of a single technique which can operate on aqueous samples in the field, rather than on dried laboratory preparations. In this perspective, we consider MP measurement technologies with a focus on both their eventual field-deployability and their respective data products (e.g., MP particle count, size, and/or polymer type). We present preliminary demonstrations of several prospective MP measurement techniques, with an eye towards developing a solution or solutions that can transition from the laboratory to the field. Specifically, experimental results are presented from multiple prototype systems that measure various physical properties of MPs: pyrolysis-differential mobility spectroscopy, short-wave infrared imaging, aqueous Nile Red labeling and counting, acoustophoresis, ultrasound, impedance spectroscopy, and dielectrophoresis.

## 1. Introduction

Microplastics (MPs) are small particles of polymer debris, commonly defined as being between 1 μm and 1000 μm [1], though no internationally agreed upon definition exists. MPs have emerged as an important subject of study for scientists with regards to their ecological impact and environmental fate and transport [2,3]. Research has been conducted to collect and analyze MPs from ponds [4], lakes [5,6], rivers [6,7], oceans [8,9,10,11], wastewater [12,13], and drinking water [14]. Studies like these inform our understanding of the extent and impact of MPs in aqueous environments. However, open questions remain about the amount and distribution of MPs in our hydrosphere, as well as the environmental and ecological impacts of these particles [15,16]. Eriksen et al. (2014) estimated the total number of plastic particles in the ocean to be over 5 trillion, weighing over 250,000 tons; however, they acknowledge that the true concentration could be higher due to the limited number and distribution of available MP datasets [15], with little data available from the southern hemisphere [17,18,19]. Recent studies have found that MPs can absorb toxic compounds and transfer these to organisms that ingest them [20,21]. Other environmental and health consequences of MPs are still being studied, but results from these investigations are limited by a lack of MP sample data, due to the shortcomings of current sampling and analysis techniques [16,22,23]. It is clear that despite being accepted as a ubiquitous presence in aqueous environments, our ability to study MPs is hindered by a lack of technologies for rapidly characterizing environmental samples for MPs.

Currently, most open-water sample collections of MPs are conducted with plankton sampling nets, which are deployed from boats and ships [20,24]. Once collected, time-intensive laboratory work, including chemical pretreatment, is commonly required to prepare MP samples for analysis [20]. The American Society for Testing and Materials (ASTM) provides standards for these MP sample collection (D8332) [25] and preparation (D8333) [26] steps, however MP analysis technologies vary considerably and tend to be researcher-specific [27,28,29].

To provide a more thorough characterization of the MP pollution in aqueous environments (e.g., lakes, ponds, rivers, and oceans), MP analysis techniques must transition from manual laboratory approaches to robust and reproducible technologies that are well-suited for measurements in the field. Development of field-portable sensors will enable studies to achieve spatial coverage, sampling frequency, and time series data not possible with current techniques [3,18]. To enable development of field-deployable sensors, measurement techniques are needed that require minimal sample preparation so they can operate as in situ as possible.

To that end, this perspective identifies several measurement techniques that we have demonstrated to be potentially advantageous for application in a field-deployable MP sensor for aqueous environments. A field-portable MP sensing system will require robust packaging, portable power electronics, and other components to be ready for field operation. In this perspective, we restrict our focus to technologies that would enable a critical measurement functionality, but do not consider the full span of engineering efforts that will be needed to adapt the proposed technologies for field use, such as sample collection. Prata et al. (2019) present a comprehensive review of the current methods used for aqueous sample collection [30]. In this perspective, we refer to each analysis technology as a “measurement technique”, while we use “sensor” to describe an engineered instrument or system that incorporates one or more measurement techniques along with any collection or concentration technologies. Each measurement technique presented is characterized by the intrinsic physical property (chemical, mechanical, or electrical) it engages to distinguish MPs from their surroundings.

## 2. Framework of Field-Deployable Microplastic Sensing

This section introduces a two-part framework to evaluate the suitability of MP measurement techniques for use in field-portable sensors. The first part is a field-deployability tradespace, which establishes a set of criteria for comparing suitability for field-deployability. The second part considers the measurement techniques themselves, how those techniques are coupled to the physical properties of MPs, and the specific data products that result. For instance, in this framework, what infrared microscopy may lack in field-deployability, it makes up for in the completeness of its MP characterization, delivering data regarding MP size, prevalence, morphology, and chemical type.

### 2.1. Field-Deployability Tradespace

Development of field-portable MP sensors will require thoughtful consideration of system design trade-offs. For instance, data quality may be associated with high cost and diminished durability. Users will have differing requirements for a field MP sensor, therefore we present what we believe are a broad and pragmatic set of criteria for evaluating the field-deployability of a technology, but do not provide numerical scores for any specific technology. Users may weight the proposed criteria according to a specific application.

**Figure 1:**
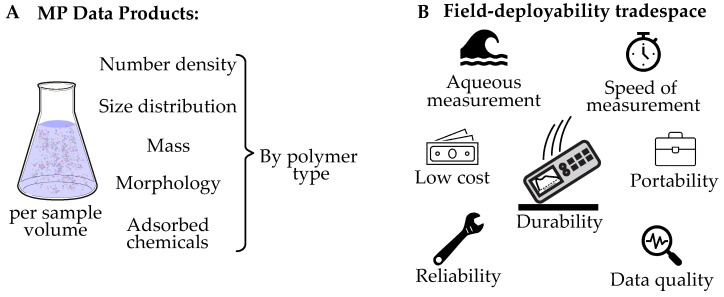
(**A**) Microplastic data products that can be collected from aqueous samples. MP number density, size distribution, mass, morphology, and adsorbed chemicals can all be further refined by measuring them by polymer type. (**B**) The key characteristics that define the field-deployable tradespace for a microplastic measurement technique for aqueous samples.

Field-deployable is defined here as having a favorable combination of the characteristics that would allow a sensor to operate in a location or on a platform where aqueous water samples are collected (e.g., on a boat, underwater vehicle, dock, or other location remote from the controlled environment of a laboratory). The characteristics we propose for consideration for a MP sensor in the field-deployability tradespace include: cost, durability, portability, low-power operation, fast-time response, and high-quality data (Figure 1). Additionally, we consider the capability to analyze aqueous samples to be beneficial insofar as it avoids the need to chemically treat, dry, or spread extracted solids on a dry surface.

### 2.2. Principles of Operation of MP Measurement Techniques and Their Data Products

Next we turn to the MP measurement techniques themselves, categorizing their principle of operation as chemical, mechanical, and electrical. For instance, techniques that rely on chemical properties are those that interact with the internal chemical structure of the sample. Many optical techniques fall in this category [31], for example, in vibrational spectroscopic analysis, a unique reflectance, absorption, or transmission spectrum is obtained for a given MP type based on internal bond structure [32]. Analysis based on mechanical properties evaluate MP size, density, modulus of elasticity, and acoustic contrast factor [33]. Electrical properties of MPs include relative permittivity, and dielectrophoretic mobility [34,35].

The most fundamental requirement of any MP sensor is the capability to positively identify MPs as polymers, as opposed to other non-MP particles in the environment, such as plankton, inorganic particles, and marine snow. This capability allows quantification of MP number density per sample volume. Ideally this number density could be broken down further by MP size distribution, polymer type, and morphology. Another requirement is the need to quantify the mass of MP particles in a sample, which can be derived from some combination of these measurements. Figure 1A illustrates the MP data products that can be collected from an aqueous sample. For completeness, we include identification of adsorbed chemicals as a MP data product, though we do not study this in our technology demonstrations. Separate studies have addressed the measurement of adsorbed chemicals in MPs [36,37,38]. Combinations of these data products can be collected and analyzed to meet the varied objectives that exist for a MP study.

Table 1 compares a selection of prominent existing MP sensing technologies with several new or early-stage technologies discussed at length within this article. The table considers each technology within the framework we have discussed, and describes notable features that are relevant to our framework. The measurement techniques are categorized by the basic properties they discern and are listed with the MP data products that they can assess/quantify/measure/observe (particle count and size, polymer type or relative mass of mixed polymer types). The shaded rows indicate the measurement techniques that we have prototyped and are described further in this perspective.

Considering both the deployability trades (Figure 1) along with performance trades (Table 1), suggests that while FTIR may not be readily field-deployable, it is one of the few techniques that generates quality data of three desired data products (polymer type, size, count). Perhaps this is why FTIR and its vibrational spectroscopic analogue, Raman spectroscopy, are the most widely used on samples collected for the laboratory and yet few studies show these methods performing in the field.

It is likely and perhaps inevitable that more than one technique can be combined to create a system which captures multiple MP characteristics for a specific application. For example, in recent studies, Raman spectroscopy, which is normally performed on dry samples, was combined with optical trapping and microscopy to perform chemical identification and sizing of MPs in seawater [65,66]. In the study of micro-particles and organisms, microscopy is often coupled with particle sorting and focusing technologies, for example, the FlowCAM couples flow cytometry with microscopy for precise imaging of micron scale specimens [80]. The combinations are numerous, and we do not explore every possible way MP measurement techniques can be combined to target multiple MP characteristics, but rather present them individually to motivate any combination of interest.

Of the techniques presented in Table 1, we chose to evaluate the following techniques: pyrolysis—gas chromatography/differential mobility spectroscopy, short-wave infrared multispectral imaging, fluorescent dyeing and imaging with Nile Red, acoustophoresis, ultrasound, impedance spectroscopy, and dielectrophoresis. These techniques were selected because they each show potential in their combination of data products and field-deployability characteristics, while having few published demonstrations as portable MP sensors. In this perspective, we report results from development efforts by Draper in conjunction with US EPA Region 9 and independent efforts at WHOI that led to prototypes and assessments of the techniques. Pyrolysis—gas chromatography/differential mobility spectroscopy, short-wave infrared multispectral imaging, Nile Red dyeing and imaging, acoustophoresis, ultrasound, and dielectrophoresis were investigated at Draper and impedance spectroscopy was investigated at WHOI.

## 3. Technology Demonstrations

### 3.1. Chemical Measurements

#### 3.1.1. Pyrolysis—Gas Chromatography with Differential Mobility Spectroscopy (Py-GC/DMS)

Pyrolysis—gas chromatography with differential mobility spectroscopy (Py-GC/DMS) is a sensing technique pioneered by Draper Laboratories. Prior to our work, this technology has been applied as a highly sensitive breath diagnostic and air quality device but, to the best of our knowledge, never to plastics analysis [55]. The closely named but distinct and older technology, pyrolysis—gas chromatography with mass spectroscopy (Py-GC/MS), has been used for identifying polymer types and has been applied to MP analysis [39,40,81].

Pyrolysis is a process in which materials are decomposed by applying heat in an inert atmosphere; applied to plastics it creates a vapor of fragments unique to the polymer type [39,82]. In Py-GC/MS, the pyrolysis products are introduced into a gas chromatograph, where the degraded fragments are separated by certain physical properties, mainly size and polarity. The separated fragments are detected using a mass spectrometer, which measures the mass of the fragments, producing a unique plot of abundance versus time [39,83]. Fischer et al. (2017) designed a Py-GC/MS system capable of unambiguously identifying seven polymer types [40]. Matsui et al. (2020) have presented an algorithm for identifying the composition of heterogeneous mixtures of polymers, successfully identifying four polymer types from an ocean water sample [41]. In terms of what MP characteristics this technique measures, it has been mostly explored for plastic type identification, but has shown potential for relative mass quantification of polymer types in heterogeneous MP samples [40,42,43,44,84].

While Py-GC/MS is a robust technique for distinguishing MP type, gas chromatography mass spectrometers are expensive (ca. $100k) [85], large, and require long (>1 h) integration times [40], making GC/MS best suited for laboratory analysis. In the spirit of field-portability, Zhang et al. (2020) have demonstrated rapid (5 min analysis time) MP identification and mass quantification with a portable mass spectrometer and custom-built pyrolyzer [44]. The portability and fast-time response of this technique demonstrates its potential for use in the field [44].

Leveraging previous work in Py-GC/MS for MP analysis, we investigated the use of Py-GC/DMS as an approach more amenable to field applications. Mass spectrometry can require large high-vacuum chambers for operation, where as DMS sensors can be miniaturized and operated under ambient conditions making them amenable to field applications.

**Figure 2:**
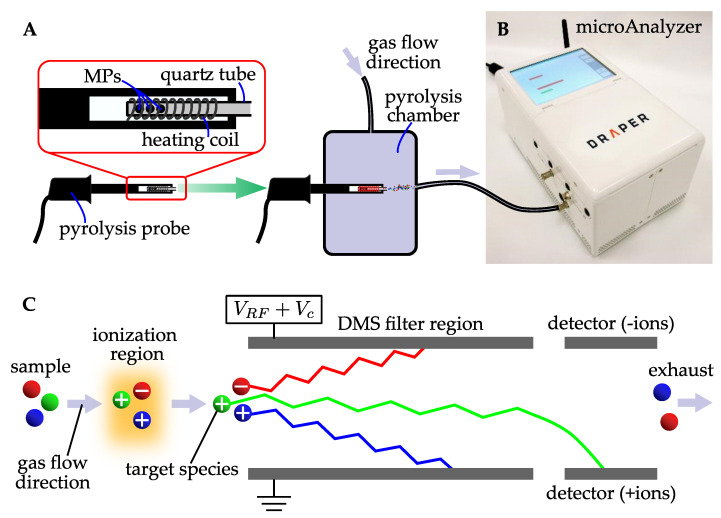
(**A**) General work-flow and instrumentation for the Py/GC-DMS study. The MP sample (prepared in a quartz tube) is placed into the pyrolysis probe attached to the pyrolysis chamber. The probe is inserted into the CDS 1500 interface unit. The interface unit is connected to the sampling inlet of the microAnalyzer in (**B**). (**C**) A schematic illustration of the DMS principle of operation. A radiofrequency ($V_{RF}$) and constant electric field ($V_{C}$) are applied across parallel plates, filtering ions based on their mobility. In this illustration, the green ion, labeled “target species”, travels without contacting the parallel plates and is detected at the positive ion detector.

In DMS, a radiofrequency and constant electric field are applied between two parallel plates. This superposition of fields causes ions to become spatially separated based on their mobility. Ions that come in contact with the detector plates are neutralized and subsequently not detected by an electrometer; ions that travel without contacting the plates are detected. At fixed compensation voltages (V${}_{C}$), certain ions will travel through the plates whereas others are absorbed, effectively making the sensor function as a tunable ion filter (Figure 2). For DMS measurements in this approach, a sweep of compensation voltages is performed to create 2-dimensional plots, which are represented as heat maps. At a fixed compensation voltage, plotting ion intensity at the electrometer (V) versus retention time creates plots similar to traditional GC chromatograms [55]. To explore Py-GC/DMS for MP analysis, we used a microAnalyzer developed by Draper Laboratories (Figure 2B), which is a portable, shoe-box sized system (10${}^{\prime \prime }$ × 6${}^{\prime \prime }$ × 5.2${}^{\prime \prime }$) weighing 6 lbs. The microAnalyzer combines three integrated technologies: a preconcentrator, a gas chromatography (GC) column, and a DMS sensor. The microAnalyzer technology has been used in many application areas, including air quality assessment on the International Space Station [86,87] and infectious disease diagnostics [55].

**Figure 3:**
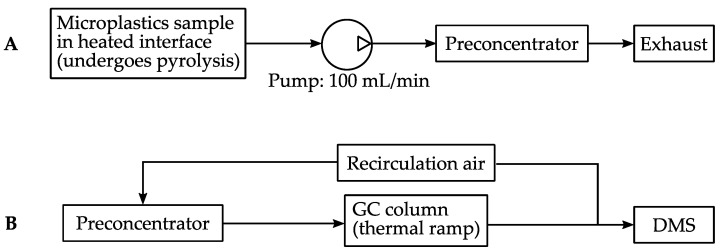
Block diagram showing typical microAnalyzer operation, using the microplastics application as an example. (**A**) MP samples are collected and adsorbed onto a preconcentrator. Sampling time can be controlled via pump duration. (**B**) After sampling, the preconcentrator is heated, releasing the sample; the sample travels through the GC column (with ambient air as carrier gas), and eluted compounds are detected via the DMS sensor.

A standardized Py/GC-DMS protocol was developed to validate whether the microAnalyzer could be used to characterize pyrolyzed MP samples, illustrated in a block diagram in Figure 3A,B. As a pyrolysis front end, we used a CDS Pyroprobe 2000 (CDS Analytical, Inc., Oxford, PA, USA) where the typical GC interface component (CDS 1500) was reconfigured to interface with the sample inlet of the microAnalyzer. The CDS 1500 interface component was maintained under a nitrogen atmosphere (200 mL/min flow) at 300 °C (Figure 2A).

MP samples were loaded into quartz sample tubes, which were placed into the CDS probe (the quartz tubes fit over the coil prior to heating). The probe was inserted into the CDS 1500 interface, at which point the microAnalyzer pump (100 mL/min) was activated for sampling. After 10 s of sampling, the pyrolysis program was initiated (200 °C or 1 s, ramped at 10 °C/ms to 700 °C, held at 700 °C for 10 s). The microAnalyzer sample pump was maintained at 100 mL/min for a total of 30 s from process start to finish.

To analyze the collected sample, a standard analysis method was initiated. The sample was desorbed from the preconcentrator via flash heating (40 °C to 300 °C within 1 s, held at 300 °C for 1 min). The sample components were separated via flow through the GC column (DB-5ms, 15 m × 0.25 mm × 1.4 μm). The GC temperature program was as follows: initial 40 °C for 2 min, ramped to 120 °C over 10 s; held at 120 °C total of 30 min. Prior to analyzing each MP sample, “blank” measurements were carried out by loading quartz tubes packed with only glass wool into the probe/interface and running the Py/GC-DMS method described above.

**Figure 4:**
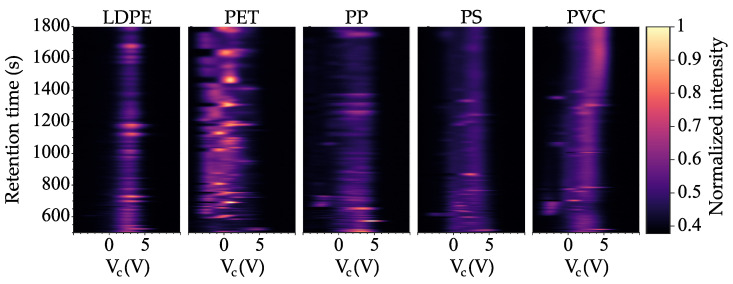
Representative Py/GC-DMS data for five plastic samples, LDPE, PET, PP, PS, and PVC. Compensation voltage (V${}_{C}$) is shown on the horizontal axis and ion intensity at the electrometer (V) is represented with the headmap. Distinct signatures are observed for each plastic.

Five different plastic samples were measured: low-density polyethylene (LDPE), polyethylene terephthalate (PET), polypropylene (PP), polystyrene (PS), and polyvinyl chloride (PVC). LDPE, PP, PS, and PVC were purchased from MilliporeSigma (Burlington, MA, USA), and PET was purchased from Goodfellow Corporation (Lindon, UT, USA). Each plastic type had a distinguishable DMS pattern (Figure 4). Additional data collection for representative plastic samples could lead to algorithms being generated for rapid polymer type identification. While testing was not conducted on sediments or other organic matter, differentiation of these contaminants from MPs is possible with Py-GC/MS and we expect that Py-GC/DMS would perform similarly [88,89].

**Figure 5:**
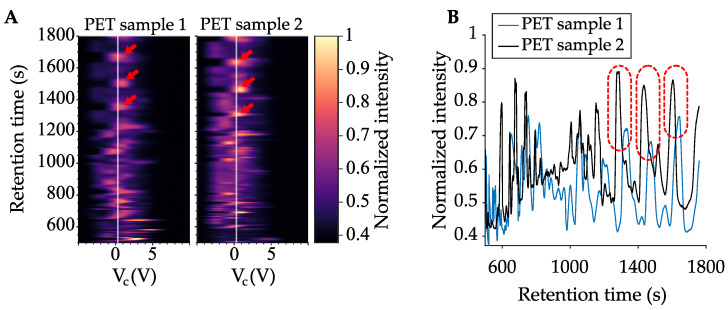
(**A**) Representative Py-GC/DMS data for two PET samples analyzed 3 months and 15 days apart with the same system, between which the system was used on other samples. (**B**) Ion intensity (V) at the electrometer is plotted versus retention time at a constant V${}_{C}$ [V${}_{C}$ = 0.3 V, drawn as a white line in (**A**)]. Three peaks are circled in red and show the correspondence between samples. Red arrows point out the same peaks in the left plots.

Moreover, repeated measurements from one plastic type (PET) indicate that the pyrolyzed plastics generate reproducible profiles (Figure 5). The two PET samples show similar profiles with a slight retention time offset, most likely due to environmental conditions. Additionally, the two samples had different amounts of input PET MPs, which is why the intensities differ slightly between the two samples. The microAnalyzer was placed in a chemical fume hood, where air currents can introduce cold spots onto the column, which can lead to slight variation in retention times. This variation can be corrected by better isolation of the column or by environmental isolation of the microAnalyzer unit. In future use, retention time offset could easily be modeled and corrected using an automated chemometric analysis. The retention time offset is clearly seen in Figure 5B, where retention times are plotted at a constant V${}_{C}$.

Our results suggest that Py-GC/DMS could be used to identify polymer types in an MP sample, provided that methods are developed to identify individual polymers within complex MP polymer mixtures. The data in Figure 4 indicate that each polymer has unique data features that do not significantly overlap; the data could be processed for pattern recognition of polymer sample data from independently pyrolyzed samples. A further, important refinement of Py-GC/DMS data analysis would be to measure the relative quantities of each polymer that is detected. In DMS, the intensity of detected ions depends on the amount of material pyrolyzed. Therefore, we are optimistic that relative quantities, and perhaps absolute quantities of MPs can be measured by calibrating the instrument using samples of known mass and polymer type. One limitation of Py-GC/DMS is that the count or size distribution of individual MPs is lost completely during pyrolysis; it would be necessary to combine this method with a front-end method that yields the complementary count/size data, such as a method that provides count of MPs without identification of polymer type, such as an impedance spectroscopy, Nile Red, ultrasound, or acoustophoresis-enabled counting method.

For use in the field, the small size, durable package, and low-cost of DMS relative to more traditional laboratory-based MP measurement techniques would make Py-GC/DMS a promising technique for future development into a portable MP sensor. One drawback is that MP sample drying is required for this technique and, as shown in Figure 5, sample analysis can take up to 30 min, which could pose constraints on certain field-use cases. Further engineering work is needed to automate the sampling and pyrolysis stages of Py-GC/MS, specifically to dry aqueous MP samples and introduce them into the pyrolyzer.

#### 3.1.2. Short-Wave Infrared (SWIR) Multispectral Imaging

Like Raman and FTIR analysis, the unique short-wave infrared (SWIR) reflectance signatures of plastics can be used to distinguish and characterize MPs in a sample [56]. A hyperspectral image reveals the spectrum (SWIR, near-infrared, Raman, or FTIR depending on equipment) at each pixel of an image, allowing particle abundance counting and classification [52]. Hyperspectral imaging has been performed on MPs for classification with Raman, FTIR, and SWIR spectroscopy. Full hyperspectral imaging is often not necessary for condensed phase spectroscopy due to the breadth of the absorption bands. As an alternative to hyperspectral imaging, it has been demonstrated that multispectral imaging can suffice, so long as wavelength bands are selected to target key features and to draw contrast between plastics and background without dwelling on wavelengths that are not useful in this determination. Multispectral imaging techniques can be implemented spectrometer-free with a low-cost camera and filters.

**Figure 6:**
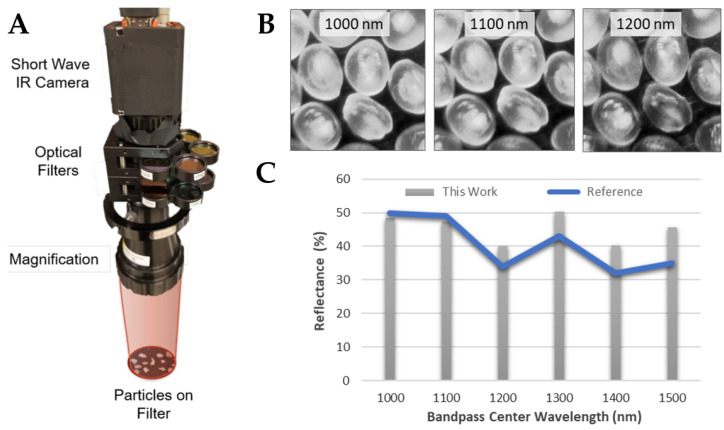
(**A**) Draper’s SWIR MP sensor setup. (**B**) Filtered images of the same field of PP MP at three bandpass wavelengths. Their individual reflectances are captured by the SWIR camera. (**C**) Comparison of the measured reflectance from a single particle at six bandpass wavelengths against the reflectance spectrum shown in Masoumi et al. (2012) [90], showing strong agreement.

We developed a multispectral SWIR technique for imaging dried seawater samples for MP detection. A SWIR camera (Sensors Unlimited Inc., Princeton, NJ, USA, Model GA1280JSX) was integrated with a wheel of infrared filters (Thorlabs, Newton, NJ, USA) to interrogate different wavelength regions. Images of a target surface were collected through each filter and compared (physical system platform is shown in Figure 6A). The filters were selected in 50 nm increments from 1000 nm–1550 nm and each has a narrow bandpass of 10 nm–12 nm. A low-cost illumination source was used to reflect SWIR light from the MP samples (source not shown in Figure 6). The illumination source provided natural discrimination from background SWIR radiation.

Basic test data were taken of a group of nominally identical PP particles (Figure 6B). The contrast between frames in Figure 6B is evident without any processing. PP has two prominent absorption peaks in the range covered by the filter set, one at 1200 nm, another at 1400 nm [90]. This manifests as a more translucent (less reflective) appearance at these two wavelengths, indicating that multispectral analysis is successful for this low-cost method. Moreover, the absorption/reflectance features have linewidths that are in general broader than the bandpass of each interference filter, providing some immunity against missing a feature. Measurement of the reflectance intensity of one particle at six bandpass wavelengths showed strong agreement with the expected reflectance spectrum of PP (Figure 6C).

**Figure 7:**
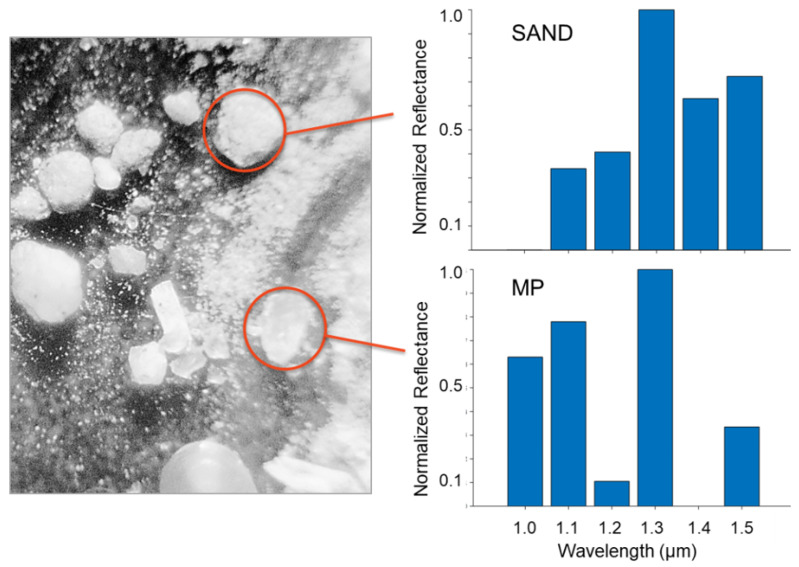
A surface with both MP and sand is imaged with the six SWIR filters. The relative intensity of the sand (**top circle**) and plastic (**bottom circle**) are distinct for the different SWIR filters.

Advancing to the more relevant case of MP in the presence of other marine particles (such as sand or biology), the method only has utility if the analyzed MP images have unique absorption spectra from background particles, and, ideally, from other types of MP. Figure 7 shows the resulting images of one surface with both PP MPs and ordinary sand. Comparing the intensity of particles at six reflectance wavelengths shows that the MP have markedly different reflectance than the sand particles (Figure 7). The histogram spectra are normalized such that reflectance at 1300 nm is unity. Once again the MP spectrum exhibits the characteristic dips at 1200 nm and 1400 nm.

**Figure 8:**
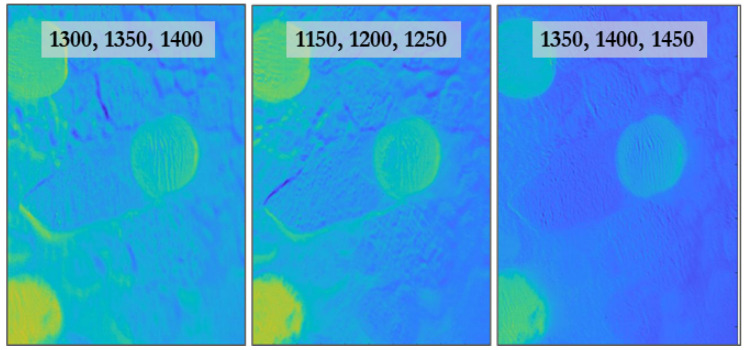
Each panel shows processed images of PP MP where the reflectance contrast at SWIR active wavelengths is drawn from neighboring wavelengths where absorption is lower. The three wavelengths listed in a panel each correspond to a filter. The middle wavelength is the reflectance value that is compared to the other two using a modified band depth analysis [57]. For instance, the middle panel shows reflectance strength at the key 1200 nm wavelength as contrasted to that of neighboring 1150 nm and 1250 nm wavelengths. The green-yellow color indicates a stronger absorption feature (lower reflectance) while the blue indicates a lack of feature presence.

When processing, it is helpful to separate MPs from the background and from one another. This can be accomplished by analyzing the reflectance values at certain key wavelengths referenced to the reflectance of adjacent, less active wavelengths. Figure 8 shows the results of this Band Depth Parameter analysis approach [57].

Note that while absorption peaks at both 1200 nm and 1400 nm are common to other MPs, for instance PS and PE, MP types can still be differentiated from one another based on the relative band gap and bandwidth. To illustrate how band depth analysis of an image can be conducted, note that the two rightmost panels in Figure 8 are not equivalent despite there being absorption at both 1200 nm and 1400 nm. This is because the 1400 nm feature is broader and therefore less image contrast is apparent versus 1350 nm and 1450 nm, the center wavelengths of its neighboring filters. The differences in spectral breadth at 1200 nm and 1400 nm are evident in the reference spectrum of Figure 8. Similarly, MPs could be differentiated from each other not only based on the center frequency of the absorption but the relative depth and width of these features as well.

Looking ahead, this sensing/referencing system could be implemented with a machine learning approach through the development of training sets from labeled images. Further experimentation should evaluate the effect of water or biofilms, as well as particle size and any size-dependent collection efficiency differences.

Based on its robustness and low cost, multispectral SWIR imaging is a technique that is inherently favorable to field deployment. Considering the data products that can be generated (MP count, size, and polymer type), it is also a fairly complete measurement technique with strong immunity from background. However, like, Py-GC/DMS, this technique still requires a dried collection of particles, increasing the total sample measurement time, requiring preparation steps that may be cumbersome in the field. Additionally, because SWIR imaging is a surface measurement, the effects of biofilms may interfere with analysis and necessitate sample cleaning, requiring further sample pre-treatment. However, the many positive factors make this technique warrant further work for fieldable sensor development for appropriate applications.

#### 3.1.3. Nile Red

Nile Red (NR) is a lipid-soluble, fluorescent dye that selectively adsorbs onto MPs. Several researchers have investigated NR staining as a technique to label and identify MPs in filtrate from environmental water samples [59,60,61,62]. To date, researchers have followed the same general NR test method: a sample is collected and chemically pre-treated to remove organic matter, then dried, stained with NR dissolved in a solvent, and finally imaged. A camera is used to image the dried material, using a filter to exclude the excitation wavelength. MPs are then identified by their bright fluorescence in the recorded image.

The published methods for NR-based detection have several notable advantages for field sensing: the equipment to perform the assay is very inexpensive, portable, and easy to operate. However, the technique requires a lengthy sequence of manual operations, including chemical handling, followed by manual image analysis. Work by Maes et al. (2017) suggests that MPs can be identified from within a matrix of natural particles, since the MPs selectively absorb more NR than the natural material [61]. However, recent work by Stanton et al. (2019) indicates that NR tends to overestimate MP abundance when samples contain other natural materials [64]. NR staining methods are a recent innovation for MP detection, and further work is needed to understand the limitations of this method. However, if chemical pretreatment is needed to remove organic material, NR-based detection would be less suitable for field testing. NR has low toxicity, but solvents containing the dye are not suitable for discharge into the environment, and must be transported from the test site for proper disposal, which is inconvenient.

**Figure 9:**
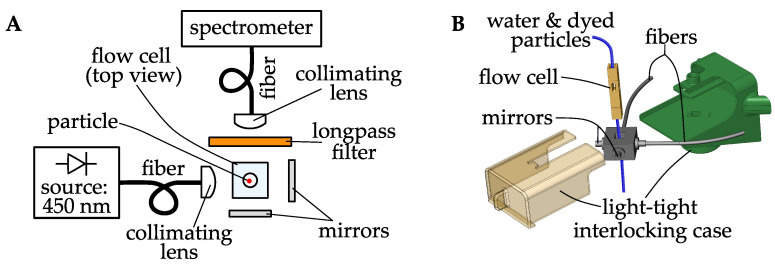
(**A**) An optical system designed to record spectra of NR-dyed particles in suspended in water. Sample water containing NR-dyed MP particles is illuminated by a 450 nm light-emitting diode and spectra are continuously recorded with a spectrometer. Spectral analysis can then be performed to determine any detections of MPs and potentially their polymer types. (**B**) The physical implementation of (**A**) is shown. The interlocking case provides portable housing for the system.

We constructed a system to accelerate an NR-based MP assay by reducing the manual actions needed to process a sample. The system operates on aqueous samples, such as rinse water from a filter. Sample water is poured into a tank. A metering pump injects NR dye dissolved in a solvent to stain particles in the liquid, and a dilution pump adds water to dilute the suspension. A valve opens to drain the tank through a flow-through fluorometer. The sample water flows slowly through a capillary tube, and is illuminated by a 450 nm, light-emitting diode. An optical fiber collects light from the capillary, and is positioned at 90° to the excitation light source to avoid direct illumination or forward-scattered light from the source. A 600 nm longpass filter is placed between the collection fiber and the capillary, to exclude light from the illumination source. The collected light is conveyed by an optical fiber to a spectrometer, which continuously records spectra in the visible to near-infrared range (300 nm to 750 nm). Usually an ordinary camera with filters is used to image NR-dyed MP samples. The proposed use of a spectrometer in our system is two-fold. First, the spectra can be post-processed to identify spectral peaks at 500 nm to 700 nm that are associated with passage of an NR-dyed MP through the fluorometer capillary. Second, analysis of spectral features could be used to identify MP polymer type. Optical system and sensor housing are shown in Figure 9.

We have operated the system on a shipboard environment for several days to verify robustness of the hardware arrangement, and have recorded spectra from PE and PS MPs placed in the fluorometer. Further testing would be needed to evaluate the performance of the system in comparison to the published methods for NR-based detection, but our initial experiences with the apparatus led us to believe that NR-based MP measurement techniques could be made more convenient for field use with the benefit of automation.

An automated NR system like this one can collect MP count and potentially polymer type, but not particle size, so for more thorough analysis it would be necessary to combine the method with additional techniques that can identify MP size. The technique is robust and portable, however, the need for consumables is disadvantageous for use in the field and the production of good data quality needs to be verified. Finally, while our work uses NR, an alternative fluorescent probe, pyrene, has recently been used for MP detection [63]. Costa et al. (2021) present a method to fluorescently detect PS MPs on salt and sand surfaces with pyrene, finding that the pyrene fluorescence intensity increased linearly as a function of MP concentration [63].

### 3.2. Mechanical Measurements

#### 3.2.1. Acoustophoresis

Acoustophoresis is a method to manipulate particles suspended in fluid using acoustic forces, and has been widely used in biomedical research to sort mixtures of blood cells and other biological microparticles [91]. In a common device configuration, a microfluidic channel is vibrated by a high frequency transducer, which results in a standing pressure wave within the channel. A particle suspended within the channel is subjected to a force from the pressure gradient, and is directed either toward or away from the nodes of the standing wave. A fundamental parameter in acoustophoresis is the *acoustic contrast factor*, Φ, which is a function of the density and compressibility of a particle, as well as the density and compressibility of the fluid surrounding the particle. The sign of Φ determines whether a particle will be directed toward (+) a pressure node or toward (−) a pressure antinode. The magnitude of the acoustic force depends on both Φ and a particle’s volume [92]. If particles are sufficiently distinct in size or acoustic contrast factor, acoustophoresis can cause particles to separate into independent streams within the channel.

Acoustophoresis can separate particles from a heterogenous suspension, but additional methods are needed to count or further analyze the content of separated particle streams. We believe acoustophoresis has potential to enable a critical particle discrimination function in an MP sensing system, provided that it can be paired with an appropriate analytical stage. There are a range of mature technologies that could be adapted for counting particles in a separated fluid volume, such as a Coulter counter, flow cytometer, or liquid laser particle counter. Several counting techniques have been miniaturized for integration into a microfludic device [93].

Prior work on acoustophoresis has focused predominantly on biomedical applications, and a large number of studies in that field have used spherical polymer MPs to test and calibrate microfluidic acoustophoresis systems [33]. With regard to environmental applications, Akiyama et al. (2020) proposed the use of acoustophoresis to separate MPs from laundry machine effluent [74]. The authors used a piezoelectric transducer to actuate a glass microchannel. Acoustic focusing of PS microspheres toward the center of the microchannel was demonstrated with 99% efficiency. Acoustic focusing of Nylon 6 microfibers and PET microfibers toward the center of the microchannel was demonstrated with 99% efficiency and 95% efficiency, respectively. Matyikiv et al. (2020) proposed acoustic separation for analysis of MPs in drinking water, and simulated the performance of an acoustophoretic device in separating using finite element analysis software [75].

**Figure 10:**
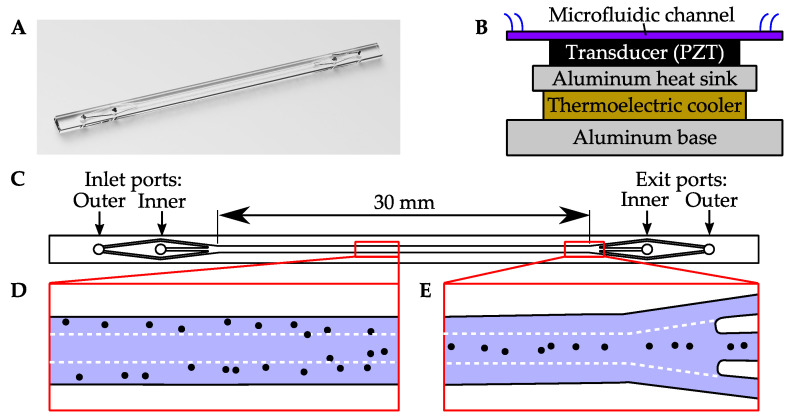
An acoustophoresis device used to test separation of MPs (**A**) A computer rendering of the PS microchannel. (**B**) A schematic diagram of the microchannel mounted to a transducer and cooling apparatus. (**C**) A top view of the microchannel, showing the trifurcated inlet and outlet ports (**D**) A section of the channel that illustrates acoustophoretic focusing of particles during co-flow operation. Particles are diverted from an outer stream toward the center of the channel. The white, dotted lines indicate the approximate boundaries of adjacent fluid streams. (**E**) Toward the end of the channel, particles are shown in the center of the channel, exiting the inner exit port.

To the best of our knowledge, acoustophoresis has not been experimentally investigated for use in analyzing MP content in environmental samples. This paper reports results from initial experiments with suspensions of two types of MPs, PE and PS. The samples were tested in an acoustophoresis device shown in Figure 10. The device comprises a PS microchannel, shown in Figure 10A that is mounted to a transducer, shown schematically in Figure 10B. The transducer is a piezoelectric crystal slab made of lead zirconate titanate (PZT), which is actuated by the amplified output of a function generator. In all experiments described below, the transducer was actuated at a frequency of 620±10 kHz. Further details on the apparatus and its electrical instrumentation are described by Lissandrello et al. (2018) [94] and Mueller et al. (2013) [95].

The geometry and operation of the microchannel are as follows: the microchannel’s cross-section is 550 μm wide by 254 μm deep, and the length of channel is 30 mm. The inlet and outlet of the microchannel are trifurcated, as shown in Figure 10C, to enable co-flow operation. In co-flow, two separate fluid streams enter the device through the inner and outer inlet ports. Flow to the inner inlet port is guided to the center of the microchannel, and flow from the outer inlet port is divided into two streams, which flow along the outer edges of the microchannel. By maintaining laminar flow conditions, the inner and outer streams do not turbulently mix. In co-flow, particles in the outer stream can be diverted into the center stream by acoustophoresis, or vice versa. Figure 10D,E illustrate diversion of particles into the inner stream. The microchannel has a trifurcated exit, which enables the contents of the center and side streams to be extracted through separate exit ports.

In a first experiment, we separated PE MPs from a water suspension using a co-flow configuration. A suspension of 20 μm diameter fluorescent PE microspheres (ThermoFisher Scientific, Waltham, MA, USA, FluoSpheres) in deionized water at a concentration of 1 × 10^5^ microspheres/mL was pumped through the outer inlet port at 100 μL/min using a syringe pump. This flow represented a water sample from which MPs were to be extracted into a flow of purified water. The purified water was prepared by passing deionized water through a 0.22 μm filter, which was then pumped through the inner inlet port at 100 μL/min using a syringe pump. The transducer was actuated at 34 V. The voltage was selected to provide a strong focusing force while avoiding overheating of the channel.

The contents of both inner and outer streams were collected in separate vials from the exit ports, respectively. In each trial, approximately 500 μL was collected in each vial. The particle content of each vial was counted using a flow cytometer (ThermoFisher Scientific, Waltham, MA, USA, Attune NxT), and gating techniques were used to exclusively count fluorescing particles. A percent separation efficiency was calculated by comparing the number of PE MPs counted in fluid recovered from the inner exit port to the number from the outer exit port. In a control trial with the device off, no fluorescing MPs were counted. With voltage applied, 98% of the PE MPs were found to be diverted into the purified water stream.

**Figure 11:**
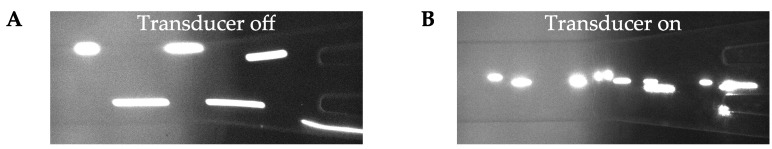
Images from a video recording of the downstream end of the microchannel, during an experiment to extract PS MPs from filtered seawater using acoustophoresis in co-flow operation. (**A**) The transducer is off, and the PS MPs are shown confined to the outer streams. (**B**) The transducer is actuated, which displaces the PS MPs to the center of the microchannel. Flow from the center of the microchannel is extracted from the inner exit port.

In a second experiment, we suspended 20 μm diameter PS spheres (ThermoFisher Scientific, Waltham, MA, USA, FluoSpheres) in Atlantic Ocean seawater. Before adding the MPs, the seawater was passed through a 0.22 μm filter to remove debris that could have clogged our flow cytometer. In this experiment, we maintained the co-flow configuration but varied the pump flow rates and actuation voltage in an effort to maximize performance of the device for separating PS MPs. The flow rate in each input was reduced to 50 μL/min to provide more time for particles to migrate toward the center of the channel. We also increased the concentration of microspheres in the test media to 1 × 10^6^ microspheres/mL after observing successful flow without clogging at this higher concentration. In a first trial, we used an actuation voltage of 46 V and observed a 96% separation efficiency. In a subsequent trial, we reduced the actuation voltage to 40 V at the same frequency and observed that the separation efficiency increased to 98%. Images from this trial are shown in Figure 11. Confirming the expected dependence on driving amplitude, further reducing the voltage to 20 V resulted in a reduced separation efficiency of 61%.

Our results suggest that PE and PS particles can be efficiently extracted from aqueous samples. The separation efficiency we reported for PS MPs largely agrees with results obtained by Akiyama et al. (2020) [74] for the same size microspheres, although our device construction and operating parameters differed. Prior work, including the experiments of Akiyama et al. (2020) [74] and simulations performed by Matviykiv et al. (2020) [75] suggest that other common polymers including PP, PET, and PVC can also be extracted with similar efficiencies. However, environmental MPs are collected in water containing a very high abundance of natural microparticles, such as plankton. A critical next step will be to evaluate the potential for acoustophoresis to selectively separate MPs from a background of natural particles. If non-MP particles can be excluded using acoustophoresis, then adding a particle counter may be sufficient to provide a count of MPs in an aqueous sample.

Separating heterogeneous mixtures of MPs by polymer from one another poses an additional, and perhaps more difficult challenge than collectively separating MPs from natural particles. Common MP polymers have a positive acoustic contrast factor [74]. If the acoustic contrast factors of particles in a heterogenous mixture share the same sign, either positive or negative, they will all focus toward the same location in the stream, the pressure node or the antinode, respectively [33,96,97].

One approach to separating two MPs that have contrast factors of the same sign in water is to instead suspend the particles in a media with a density intermediate between the two polymers so that the contrast factor for one of them becomes negative. For example, Gupta et al. (1995) separated PS and LDPE particles in a glycerol-water mixture [97], and Petterson et al. (2007) separated PS and PMMA particles of the same size suspended in water to which cesium chloride was added, and also demonstrated separation of 2 μm, 5 μm, 8 μm and 10 μm PS particles by size [73]. Acoustophoretic media exchange devices have been developed to transfer particles en masse from one fluid to another fluid, and may be useful for diverting MPs into fluids that facilitate separation by polymer type [98].

Acoustophoresis offers several potential advantages for a field-deployable MP sensing system. First, use of aqueous sample media avoids the need to extract and dry particles. Second, the forces in acoustophoresis depend on bulk, mechanical properties of materials, therefore the manipulation may be effective with MPs affected by surface fouling from biofilms or other materials. Third, acoustophoresis has been shown to be effective for separating very small particles [99,100], include sub-micrometer particles [101]. The relative abundance of small MPs is very high in comparison to MPs of 100 μm in diameter or more that are retained by surface trawling nets, so acoustophoresis may be useful for analyzing smaller samples than have previously been required.

The field-deployability of an acoustophoresis system will require selecting electronic components, such as an RF amplifier and control computer that are compact and durable. We believe that all the required electronics could be miniaturized to fit within a briefcase-size package. Our apparatus currently requires a trained operator to process samples, but we believe the steps to process a sample could be automated using computer-controlled fluidic components. A significant engineering effort would be required to fully automate sample processing, but we believe that a reliable, automated acoustophoresis system can be built.

#### 3.2.2. Ultrasound

Ultrasound imaging systems use high-frequency acoustic waves to interrogate materials. Waves are emitted from a transducer, and the intensities and time-of-flight information of reflected waves are mathematically processed to generate a depth image. Energy is reflected due to differences in acoustic impedance among imaged materials. Acoustic impedance, *z* (Pa·s/$m^{3}$), is a quantity that measures the resistance to flow in particles of the material when subjected to a local pressure, and is a function of the density ρ (kg/$m^{3}$), and bulk modulus of the material, *k* (N/$m^{2}$), of a material, where

$$z=\sqrt{\rho k}$$

The bulk modulus, *k*, is the inverse of a material’s compressibility (discussed in Section 3.2.1).

The acoustic impedance of water is approximately 1.5 × 10^6^ Pa·s/$m^{3}$. The acoustic impedances of common polymers differ from that of water (e.g., PP is 1.9 × 10^6^ Pa·s/$m^{3}$ and PS is 2.5 × 10^6^ Pa·s/$m^{3}$ [102]), and will therefore reflect or scatter ultrasound radiation, depending on the size of the particle. In general, the smallest particle resolvable by an ultrasound system is proportional to the ultrasound wavelength. Particles equal to or smaller than the acoustic wavelength act as acoustic scatterers, and appear as point sources, without discernable features.

**Figure 12:**
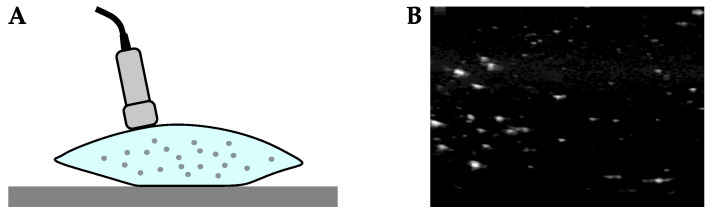
(**A**) An illustration of the apparatus used to demonstrate ultrasound imaging of MPs. PE bags were filled with suspensions of several types of MPs in tap water, and an ultrasound probe was applied to the outer surface of the bag to image its contents. (**B**) An image captured by the ultrasound machine system of PE MPs ranging from 125 μm to 150 μm in diameter.

To demonstrate ultrasound imaging of small MP particles, we filled PE bags with suspensions of MPs in tap water (Figure 12). A portable, clinical ultrasound machine (GE Healthcare, Chicago, Il, USA, Logiq-E with 12L-RS probe) was used in B-mode to record two-dimensional ultrasound images by applying the ultrasound probe against exterior of the bags. The maximum frequency of our ultrasound probe was 13 MHz, which corresponds to a spatial wavelength of approximately 26 μm.

Figure 12B shows an ultrasound image recorded of PE spheres ranging in diameter from 125 μm to 150 μm (Cospheric L.L.C., Santa Barbara, CA, USA, CPMS-0.96 125–150 μm). The diameters of these MPs are several times larger than the acoustic wavelength emitted by our probe, and are therefore visible as discrete particles. We also imaged PP, PS, PET, and PVC MPs that were obtained by grinding and sieving bulk polymer samples to be within the 125 μm to 150 μm size range. Images of these particles appeared similar to Figure 12B.

The image shown in Figure 12B demonstrates the basic functionality of ultrasound for MP imaging using a clinical scanner. Ultrasound systems are available with much higher wavelengths (and therefore spatial resolution) than the clinical system we demonstrated. However, a disadvantage of using smaller wavelengths is that penetration into the sample volume is decreased. For example, resolution of features below 1 μm has been demonstrated in solid samples using transducers operating in the gigahertz range to image micrometer-scale sample volumes [103].

In our demonstration, we acquired two-dimensional images of planes within a three-dimensional sample volume. Several different approaches could be used to analyze the contents of an entire three-dimensional sample volume. Transducers limited to two-dimensional imaging (B-mode ultrasound) can be mechanically rotated or translated to scan and obtain a three-dimensional image of a sample volume [104]. Alternatively, matrix array transducers generate three-dimensional ultrasound images using beam-steering techniques, thereby avoiding the need to scan a volume by physically displacing a transducer. Possible alternatives to scanning a fixed sample volume include scanning a flow of water as it moves past a stationary probe, or dragging a probe through a body of water. However, if the water has a high velocity relative to a transducer, a very high sampling frequency would be needed to resolve the individual scatterers.

MP particles can be counted and measured using conventional image processing techniques. However, it will be necessary to distinguish MPs from natural microparticles in water samples to produce meaningful count and size data in a field environment. The intensity of acoustic reflections is a function of acoustic impedance, and it may be possible to discriminate between MPs and other particles by intensity analysis alone. However, more advanced image processing techniques or active manipulation of the particles may be needed. For example, ultrasound elastography describes a family of techniques that combine ultrasound imaging with an additional method of mechanical excitation, such as application of a strong acoustic pulse [105], or use of a laser pulse in photoacoustic imaging [106]. Mechanical properties such as the shear modulus of a material can be measured using ultrasound elastography, which may be very helpful in distinguishing MPs from softer biological materials or very rigid silicates, for example. Classification of polymer by type using ultrasound will clearly be more challenging than discriminating MPs from natural background particles. However, common polymers do differ from one another in density and elastic modulus; so it may be possible to classify MPs by polymer type using elastography methods.

Experience with clinical instruments suggests that ultrasound technology is adaptable to field-portable MP sensing, provided that methods to distinguish MPs from natural microparticles can be identified and made field-portable. Clinical ultrasound machines are robust devices that safely and reliably generate images suitable for medical diagnoses. Battery powered, handheld ultrasound imaging devices are widely available, and are inexpensive relative to the laboratory instruments currently used to analyze MPs.

### 3.3. Electrical Measurements

#### 3.3.1. Impedance Spectroscopy

Impedance spectroscopy is a technique that can be used to infer the electrical properties of particles directly in a liquid medium [107,108,109,110,111]. In the microfluidic and biomedical fields, it is used for analysis of individual cells, e.g., discrimination of living and dead red blood cells [107,110,111]. The impedance between parallel or coplanar electrodes is measured simultaneously at several frequencies. As particles pass the electrodes, they affect the complex impedance (real and imaginary) according to their relative permittivity. At low or zero frequency, the impedance change is proportional to particle volume, and is the working principle of a Coulter counter. Impedance changes at high frequency reflect both the material properties and the size of the particle [35,107]. To distinguish between the effects of size and material type, measurements are conducted at high and low frequencies simultaneously [107,111]. Measurements are typically made using phase-sensitive electronics, such as a lock-in amplifier, and a transimpedance amplifier or a bridge circuit [107,111,112]. The data from a flow-through impedance spectrometer can be analyzed to obtain continuous measurements of particle count, material type, and size.

**Figure 13:**
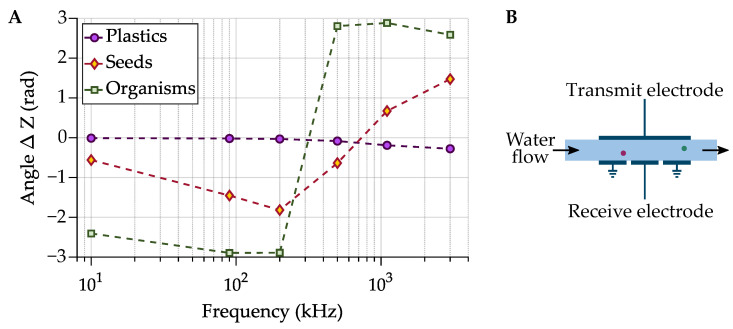
(**A**) The average angle of the impedance change associated with different particle types. The angle of seeds and organisms changes direction with frequency, whereas it remains small for plastics. (**B**) Diagram of the impedance measurement flow cell used [35].

Plastic and biological materials have different electrical properties. Biological materials exhibit an additional frequency dependence, or dispersion, due to interfacial polarization at the cell membrane [107,109,111,113]. Cells are also filled with conductive cytoplasm, and at high frequencies the electric field can pass through the membrane and the cell’s internal properties can be measured [107,109,111]. Plastics, however, are generally homogeneous and therefore do not exhibit this additional dispersion. Figure 13A shows how this difference is represented in the impedance spectra of the different particle types.

**Figure 14:**
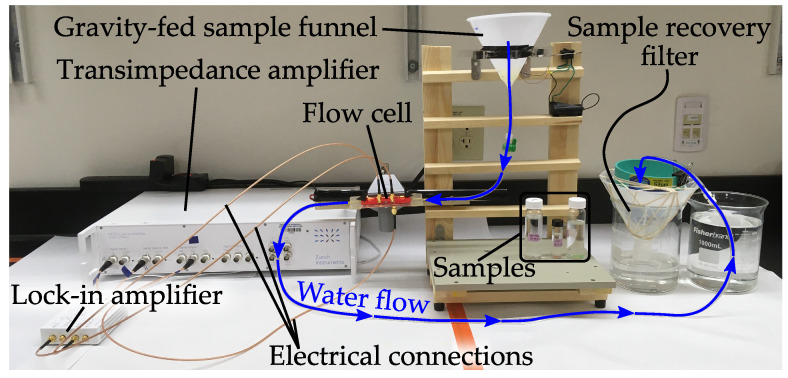
The test setup used in Colson and Michel (2021) to demonstrate impedance spectroscopy for quantification of MPs directly in a flow of water [35].

**Figure 15:**
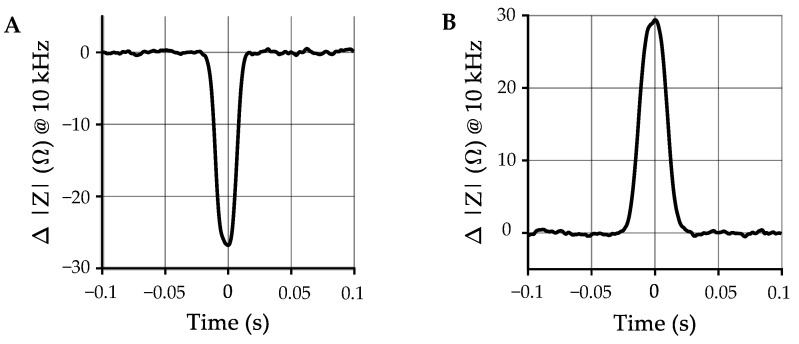
A comparison of impedance time series showing detections (**A**) a moina crustacean (570 μm–780 μm) and (**B**) PE bead (600 μm–710 μm). The direction of the impedance change for the PE bead is opposite that of the moina [35]. Time is normalized with respect to the peak.

In a recent publication, we demonstrated the use of impedance spectroscopy for flow-through MP quantification [35]. A flow channel was built from two electrode plates, shown in Figure 13B. An AC potential was applied to the ‘transmit’ electrode, and a transimpedance amplifier was used to monitor the current passing between the electrodes to the ‘receive’ electrode (physical experimental setup is pictured in Figure 14). Figure 15 shows the clear difference in impedance change between a MP sample and an equally sized organic material (in this case the crustacean moina) that were flowed through the system. We showed that MP could be robustly differentiated from biological material with a 90% recovery rate for MPs (300 μm–1000 μm diameter beads) and a 1% false positive rate for misclassification of biological material. It is noted that additional improvements are needed to cover the full MP size range (1 μm–1000 μm) [1], but the results are promising for future developments. While we did not investigate polymer type identification with impedance spectroscopy, polymers could be coarsely grouped by relative permittivity.

Impedance spectroscopy is suitable for future use in field instrumentation. It can provide MP count data and future investigations may show its ability to coarsely identify polymer type and particle size. Measurements are automated, conducted directly on a flow of water, require minimal sample preparation (a screen to prevent large particles), and the equipment is portable in size. The technique is high-throughput (>100 mL/min), especially when compared to the labor intensive processes required for standard laboratory analysis of MPs. It is especially applicable for continuous monitoring applications or for bulk sample processing. When combined with a sorting mechanism, impedance spectroscopy could be used to isolate MPs from natural materials. Once sorted, the MPs could be analyzed by other flow-through instrumentation which generate polymer type or particle size data or could be collected for future laboratory measurements.

#### 3.3.2. Dielectrophoresis

Dielectrophoresis (DEP) is a phenomenon in which a force acts on a dielectric particle subjected to a non-uniform electric field, causing the particle to move. The motion may be either in the direction of an increasing or decreasing field gradient, depending on the relative permittivities of both the particle and medium that it occupies. The magnitude of the force also depends on the relative permittivities of the particle and medium, as well as the characteristics of the applied electric field.

DEP is frequently applied in biomedical research to sort and manipulate cells and other biological microparticles suspended in fluid. DEP devices typically use electrodes that are patterned onto a wall of a microfluidic channel or microwell to selectively trap or divert particles into collection channels based on their physical properties. The selectivity of the process depends on the electrical and mechanical properties described above, as well as the hydrodynamic properties of the particles and medium (e.g., the drag coefficient of a particle, and viscosity of the medium).

**Figure 16:**
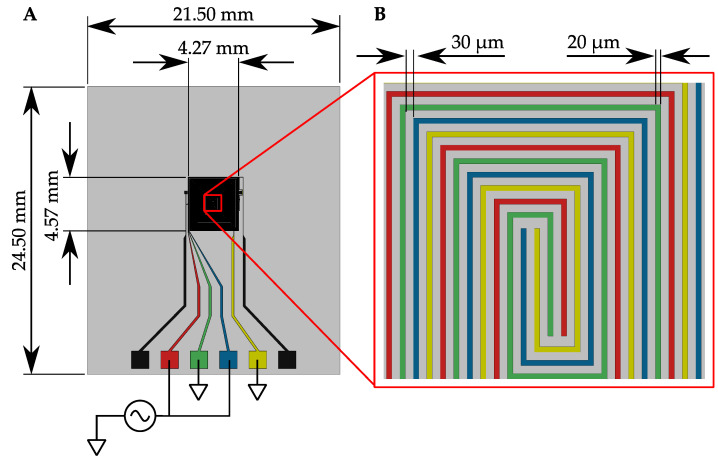
(**A**) The microfabricated DEP electrode array for MP particle manipulation in liquid. (**B**) A close up of the array’s spiral pattern and trace widths and intra-electrode spacing. The spiral pattern provides an extended surface for capturing particles in the liquid.

Figure 16 shows a microfabricated electrode array for DEP particle manipulation. The array is comprised of copper traces that were deposited on a glass substrate using a lithography process. Silicon nitride was then deposited on the device to act as an insulator. The copper traces form a spiral pattern, which provides an extended surface for capturing particles from a volume of liquid in which the device is immersed. Non-uniform electric fields form between adjacent traces to apply DEP forces to nearby particles. The spiral is comprised of four traces, which terminate in the color-coded pads shown in Figure 16A. For the demonstration presented in this article, an alternating current power supply was connected to the devices (Figure 16A), which causes each adjacent electrode to be of opposite polarity to the electrode immediately adjacent to it. Representative trace widths and intra-electrode spacing are shown in Figure 16B.

**Figure 17:**
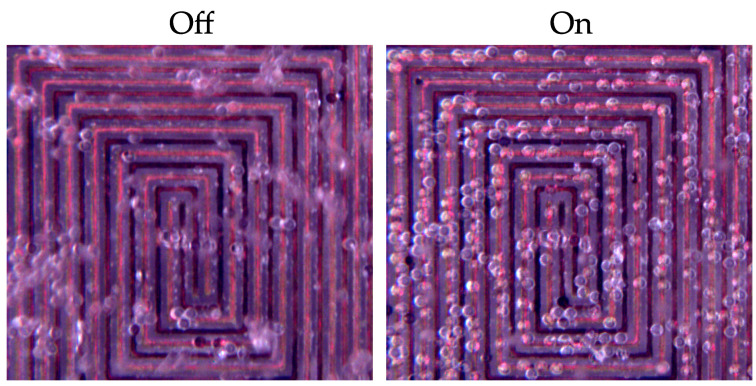
Microscope images of MP spheres over the DEP electrode array. When no voltage is applied, the MPs are suspended above the device. When voltage is applied, the MPs are attracted to the array.

Figure 17 shows microscope images that demonstrate trapping of MP spheres using our DEP electrode array. The array was placed at the bottom of a cylindrical well and then covered by a fluid comprised of 48 μm PS spheres (PSMS-1.07 38–48 μm, Cospheric L.L.C.) suspended in deionized water. In Figure 17, the device is shown with no voltage applied (off), in which MPs remain in suspension above the device. When the device was turned on by applying 40 V at 10 kHz, spheres near the array were immediately attracted to it, and adhered to the array while the voltage was sustained.

DEP has been widely used in microfluidic devices to manipulate liquid suspensions of spherical MPs for the purposes of testing and calibrating devices that are typically intended for biomedical applications [114,115]. Recently, Bu et al. (2020) proposed to sort and separate MPs from bodily fluids using DEP [78]. However, to the best of our knowledge, DEP has not been adapted for sensing MPs in aqueous environmental samples.

The relative permittivities of common polymers range from approximately 2 to 4 at low frequencies. In contrast, water has a relative permittivity of approximately 80, depending on salinity, temperature, and other variables. The stark difference in relative permittivity between common polymers and water suggests that DEP may be efficacious for diverting MPs from a flow of water and water-based organisms, since the magnitude of DEP forces depends on the contrast in relative permittivity between a particle and the surrounding medium. However, misidentification of particles that have similar permittivities to those of common polymers, such as chitin or other natural biopolymers, may pose a challenge. Operation with seawater may pose further challenges, due to the conductivity of seawater, and may require a dilution step or medium exchange device to transfer the particles to a less conductive fluid.

As with acoustophoresis, DEP as considered here is a microfluidic method for separating MPs from natural particles, and potentially sorting them by polymer type. Additional instrumentation would be needed to count, measure, or classify MPs by polymer type. A variety of microfluidic particle counting methods have been described in the literature [93], and the impedance spectroscopy method discussed above suggests that multiple electrical measurements could be acquired in a compact device with electrical measurement stages arranged in series.

DEP has several potential advantages for field sensing of MPs in aqueous samples. DEP electrode arrays have no moving parts, and thus are appealing for construction of robust and inexpensive instruments. In comparison to techniques like FTIR or SWIR, DEP and impedance spectroscopy may be useful for analysis of biofilm-coated MPs. Spectroscopic instruments are prone to misclassifying biofilm-coated MPs, since the light emitted by the instruments can be obscured by superficial biofilm. In contrast, DEP and impedance spectroscopy probe the bulk material of a particle, and may be useful in identifying MPs without a need to remove a biofilm layer. An ability to analyze samples without cleaning steps would be especially useful for a field-deployable sensor.

## 4. Discussion

Several prototypes and feasibility studies for field-deployable MP measurement techniques were performed at Draper and WHOI based on an analysis of (a) their eventual deployability and (b) the number and quality of data products produced. This work seeks to inform the MP community of the potential of these techniques in creating rapid and automated MP sensors within a framework that addresses the full scope of practical/technological trade-offs to be considered. It should be noted that, while MP particle size was discussed as various levels for each technology, with increasing concern about the pervasiveness of sub-micro and nanoplastics, technologies should invest in capabilities in these size ranges. In addition to the MP measurement techniques presented here, development of efficient, portable MP sample collection and concentration technologies is equally crucial for creating full aqueous MP sensor systems.

Rapid and portable MPs sensors would allow widespread studies monitoring at potential sources such as plastic production plants or waste water plants [116]. Technologies like these are essential for equipping scientists with the tools they need to better understand the fate, transport, abundance, and environmental impact of MPs in aqueous environments. Further, technologies like these are important for addressing the concerns of MP pollution and contamination in fisheries and aquaculture [117]. Effective, rapid, and affordable sensors are also integral in shaping and backing policies to limit MP contamination from factory run-off or keeping drinking water safe (e.g., California’s Safe Drinking Water Act) [118].

## Figures and Tables

**Table 1:** Measurement techniques with applicability to MP analysis. Techniques evaluated in this paper are shaded gray; Impedance spectroscopy was investigated at the Woods Hole Oceanographic Institution (WHOI) and the remaining techniques were investigated at Draper. ${}^{†}$ *Separation from Non-MPs* is not a data product but instead is considered an enabling technology for measuring MP data products. * This observable has only preliminary data or is currently theoretical only.

	Measurement	Data Products	Considerations for Field-Deployability
**Chemical**	Py-GC/MS	Polymer Type, Relative Mass	*Pyrolysis-Gas Chromatography / Mass Spectrometry* (Py-GC/MS). Demonstrated accuracy for MP polymer type identification (ID) [39,40,41,42,43,44]. Can characterize the identity of sample contaminants. Requires a dry sample, time-intensive data collection and is often bulky.
	FTIR Spectroscopy/Imaging	Polymer Type, Count, Size	*Fourier Transform Infrared Spectroscopy* (FTIR). Demonstrated accuracy for MP polymer type ID [45,46], often used with attenuated total reflectance (ATR) [47]. Can characterize the identity of sample contaminants. Time-intensive due to chemical pretreatment and scanning of dried sample surface. Requires expensive, precisely aligned optomechanics, often bulky. Traditionally, sample must be dry.
	Raman Spectroscopy/Imaging	Polymer Type, Size, Count	Demonstrated accuracy for MP polymer type ID [45,46,48,49,50]. Can characterize the identity of sample contaminants. Time-intensive due to chemical pretreatment and scanning of dried sample surface. Requires expensive, precisely aligned optomechanics, often bulky. Traditionally, sample must be dry.
	Hyperspectral Imaging	Polymer Type, Count, Size	Demonstrated accuracy for MP polymer type ID in near-infrared [51,52,53] or short-wave infrared [54] regimes. Can characterize the identity of sample contaminants. Time-intensive due to chemical pretreatment. Requires expensive, precisely aligned optomechanics, often bulky. Traditionally, sample must be dry.
	Py-GC/DMS	Polymer Type, Relative Mass	*Pyrolysis-Gas Chromatography/Differential Mobility Spectrometry* (Py-GC/DMS). Robust and portable package, currently used in non-MP field applications [55]. Can chemically characterize the identity of sample contaminants. Lower cost and smaller than Py-GC/MS. Requires a dry sample and time-intensive data collection. Heritage as highly sensitive breath diagnostic and air quality device [55].
	Multispectral Imaging	Polymer Type, Count, Size	Rapid sample imaging. Time-intensive due to chemical pretreatment. Uses portable and relatively low-cost equipment compared to spectrometers. Traditionally, sample must be dry. Heritage in mineral and polymer type identification [56,57,58].
	Fluorescent Dye	Count, Size	Initial demonstrations with Nile Red [59,60,61,62] and pyrene [63] in laboratory MP studies. Uses low-cost equipment (dye, camera, and filter). May not require chemical pretreatment. Potential for false positives [64]. Traditionally, sample must be dry.
**Mechanical**	Laser Optical Trapping	Separation from Non-MPS ${}^{†}$	Preliminary demonstrations of usefulness for MP identification when coupled with Raman Spectroscopy [65,66]. Performed in a microfluidic device, reducing sample preparation time.
	Photonic Optical Trapping	Size *, Separation from Non-MPs	Performed in a microfluidic device, reducing sample preparation time. Heritage in particle sorting and manipulation for bio-sensing and imaging [67,68,69,70].
	Field Flow Fractionation (FFF)	Size, Separation from Non-MPs	Centrifugal [66], Asymmetrical flow [66,71], or Thermal [72]. A recent study used FFF with Raman Spectroscopy to identify MP type [66]. Performed in a microfluidic device, reducing sample preparation time. Equipment is relatively low-cost and portable.
	Acoustophoresis	Polymer Type *, Size, Separation from Non-MPs	Performed in a microfluidic device, reducing sample preparation time. Equipment is relatively low-cost and portable. Heritage in cell and particle manipulation in microfluidics field [33,73]. Recent studies have demonstrated MP sorting [74,75].
	Ultrasound	Polymer Type *, Size *	Performed in a microfluidic device or liquid volume, reducing sample preparation time. Equipment is relatively low-cost and portable. Heritage in flow cytometry [76]
**Electrical**	Impedance Spectroscopy	Polymer Type *, Count, Size	Preliminary demonstrations of accuracy in MP identification [35]. Performed in microfluidic device, reducing sample preparation time. Equipment is relatively low-cost and portable [77].
	Dielectrophoresis	Polymer Type *, Count, Size	Performed in microfluidic devices, reducing sample preparation time. Equipment is relatively low-cost and portable. Heritage in cell and particle manipulation, some recent studies on use with MPs [34,78,79].

## Abbreviations

The following abbreviations are used in this manuscript:

ATR	Attenuated total reflectance
DEP	Dielectrophoresis
DMS	Differential mobility spectroscopy
FFF	Field flow fractionation
FTIR	Fourier transform infrared spectroscopy
GC	Gas chromatography
GC/MS	Gas chromatography-mass spectrometry
ID	Identification
LDPE	Low-density polyethylene
MP	Microplastic
MS	Mass spectrometry
NR	Nile Red
PET	Polyethylene terephthalate
PP	Polypropylene
PS	Polystyrene
PVC	Polyvinyl chloride
Py-GC/DMS	Pyrolysis-Gas Chromatography/Differential Mobility Spectrometry
Py-GC/MS	Pyrolysis-Gas Chromatography/Mass Spectrometry
PZT	Lead zirconate titanate
SWIR	Short-Wave Infrared
WHOI	Woods Hole Oceanographic Institution
